# Risk stratification for venous thromboembolism in patients with testicular germ cell tumors

**DOI:** 10.1371/journal.pone.0176283

**Published:** 2017-04-21

**Authors:** Angelika Bezan, Florian Posch, Ferdinand Ploner, Thomas Bauernhofer, Martin Pichler, Joanna Szkandera, Georg C. Hutterer, Karl Pummer, Thomas Gary, Hellmut Samonigg, Joerg Beyer, Thomas Winder, Thomas Hermanns, Christian D. Fankhauser, Armin Gerger, Michael Stotz

**Affiliations:** 1 Division of Clinical Oncology, Department of Internal Medicine, Medical University of Graz, Graz, Austria; 2 Research Unit Genetic Epidemiology and Pharmacogenetics, Medical University of Graz, Graz, Austria; 3 Center for Biomarker Research in Medicine (CBmed), Graz, Austria; 4 Department of Experimental Therapeutics, The University of Texas, MD Anderson Cancer Center, Houston, Texas, United States of America; 5 Department of Urology, Medical University of Graz, Graz, Austria; 6 Division of Angiology, Department of Internal Medicine, Medical University Graz, Graz, Austria; 7 Department of Oncology, University Hospital Zürich, University of Zürich, Zürich, Switzerland; 8 Department of Urology, University Hospital Zürich, University of Zürich, Zürich, Switzerland; Maastricht University Medical Center, NETHERLANDS

## Abstract

**Background:**

Patients with testicular germ cell tumors (TGCT) have an increased risk for venous thromboembolism (VTE). We identified risk factors for VTE in this patient cohort and developed a clinical risk model.

**Methods:**

In this retrospective cohort study at the Medical University of Graz we included 657 consecutive TGCT patients across all clinical stages. A predictive model for VTE was developed and externally validated in 349 TGCT patients treated at the University Hospital Zurich.

**Results:**

Venous thromboembolic events occurred in 34 (5.2%) patients in the Graz cohort. In univariable competing risk analysis, higher clinical stage (cS) and a retroperitoneal lymphadenopathy (RPLN) were the strongest predictors of VTE (p<0.0001). As the presence of a RPLN with more than 5cm in greatest dimension without coexisting visceral metastases is classified as cS IIC, we constructed an empirical VTE risk model with the following four categories (12-month-cumulative incidence): cS IA-B 8/463 patients (1.7%), cS IS-IIB 5/86 patients (5.9%), cS IIC 3/21 patients (14.3%) and cS IIIA-C 15/70 patients (21.4%). This risk model was externally validated in the Zurich cohort (12-month-cumulative incidence): cS IA-B (0.5%), cS IS-IIB (6.0%), cS IIC (11.1%) and cS IIIA-C (19.1%). Our model had a significantly higher discriminatory performance than a previously published classifier (RPLN-VTE-risk-classifier) which is based on the size of RPLN alone (AUC-ROC: 0.75 vs. 0.63, p = 0.007).

**Conclusions:**

According to our risk stratification, TGCT patients with cS IIC and cS III disease have a very high risk of VTE and may benefit from primary thromboprophylaxis for the duration of chemotherapy.

## Introduction

Testicular germ cell tumors (TGCT) represent one of the most curable solid malignancies as cisplatin-based chemotherapy is highly efficacious in achieving durable remissions even in widely metastatic disease [1–3]. The recent focus of clinical research in TGCT has therefore shifted on the prevention of treatment-related complications like venous thromboembolism (VTE) [4]. The risk of VTE is increased around 4–6 fold in cancer patients as compared to the general population [5,6]. Moore et al. have demonstrated a very high incidence of thromboembolic events in patients receiving cisplatin-based chemotherapy [7]. Thromboembolic events have a high impact on morbidity of cancer patients and are negative predictors of survival [8–12]. Therefore predictive factors for VTE are needed to identify subgroups with the highest risk and thus the potentially greatest benefit from primary thromboprophylaxis. Khorana et al. recently developed a predictive model for chemotherapy-associated VTE [6]. However, the validation of the Khorana model included only 39 patients with TGCT [13]. Recently, Srikanthan et al. have shown that a large (more than 5cm in maximal axial diameter) retroperitoneal lymphadenopathy (RPLN) is a strong risk factor for VTE in TGCT, and that this predictor provides a higher discriminatory accuracy for the prediction of VTE than the Khorana score. However, the study by Srikanthan included only patients with disseminated TGCT [13]. In this study, we examined the incidence of VTE in TGCT patients across all clinical stages and developed a clinical risk model for VTE in patients with TGCT.

## Materials and methods

### Subjects

All consecutive patients (n = 657) with histologically confirmed TGCT, presenting to the Division of Oncology at the Medical University of Graz between January 2000 and December 2013, were retrospectively reviewed. Patients were initially staged using computed tomographic (CT) scans of the abdomen, CT scan or X-ray of the chest and postoperative tumor markers α-fetoprotein (AFP), human chorionic gonadotropin (HCG) and lactate dehydrogenase (LDH). Patients with disseminated disease were risk-classified according to the International Germ Cell Cancer Collaborative Group (IGCCCG) classification [14,15].

Follow-up data were retrieved until January 2015. Follow-up investigations at our center were performed according to a local protocol and were adapted in 2007 and 2012 according to recent publications [16–18]. Electronic and paper medical records of all 657 consecutive TGCT patients were retrospectively reviewed and thromboembolic events were documented in our in-house administrative system. VTE was defined as symptomatic or incidental deep vein thrombosis (DVT), visceral thrombosis and pulmonary embolism (PE) and had to be confirmed by imaging such as angiography, venous doppler ultrasound, magnetic resonance imaging, computed tomography or ventilation/perfusion scan. Patients with VTE at cancer diagnosis (n = 3) were not counted as VTE events. All VTE events during the first year of follow-up were considered for the development of the VTE risk stratification rule. The validation cohort consisted of 349 consecutive TGCT patients treated at the University Hospital Zurich between January 2003 until December 2013. Patient records were anonymized and de-identified prior to analysis. The study was approved by the Institutional Review Board of the Medical University of Graz (No. 26–196 ex 13/1) and the Kantonale Ethikkomission Zurich (KEK StV-No.25-2008).

### Statistical analysis

All statistical analyses were performed using Stata (Windows version 13.0, Stata Corp., Houston, TX, USA). Fatal and non-fatal, symptomatic or incidental VTE was defined as the primary endpoint of this analysis. In both the development and the external validation cohort, the risk of VTE was estimated with competing risk cumulative incidence estimators according to Marubini & Valsecchi.(Stata routine stcompet) [19]. Cumulative incidence functions between two or more groups were compared with Gray’s test (self-written routine stgrays) [20]. Uni- and multivariable modeling of time-to-VTE was performed with Fine & Gray proportional subdistribution hazards models (Stata routine stcrreg) [21]. All these competing risk analyses considered mortality as the competing event of interest. Risk of mortality was analyzed using the inverse of the Kaplan-Meier product limit estimator, the log-rank test, and the Cox model. We evaluated the impact of VTE on mortality with a clock-forward, Semi-Markov, three-state, unidirectional illness-death model with proportional hazards for the transitions into the death state [22].

Next, we aimed to derive an empirical risk stratification rule for thrombotic risk in all TGCT patients. This rule was first developed in the Graz cohort, and then externally validated in the Zurich cohort. Model development in the Graz cohort was performed by considering the variables with the strongest association with VTE in univariable competing risk analysis combined with a subject-matter-knowledge-approach [22]. In detail, stage and chemotherapy were highly collinear. Given that stage emerged as a stronger VTE predictor than chemotherapy (as indicated by χ^2^ statistics) and showed also the highest discriminatory potential towards VTE (as indicated by Harrell’s C statistic), we decided to keep stage as a fixed variable in the model building process [23]. RPLN was included as a second variable in the risk stratification model because in a subgroup analysis of patients with metastasized cS IS–IIIC disease, the most relevant predictors of VTE were clinical stage III disease and a large RPLN. As the presence of a RPLN with more than 5cm in greatest dimension without coexisting visceral metastases is classified as stage IIC, we further subdivided our tumor stage variable and hence constructed the empirical VTE risk stratification rule with the following four categories cS IA-B, cS IS-IIB, cS IIC and cS IIIA-C. In external validation, we pre-specified to consider the model successfully validated given it achieves a comparable C-Index for discrimination and comparable absolute VTE risks in the risk groups defined by the risk model [23].

The areas under the receiver-operating-characteristic-curve (AUC-ROC) for the RPLN-VTE-risk-classifier according to Srikanthan et al. and the novel risk model were compared non-parametrically using a chi-squared test (Stata routine rocgold) [13,24]. In a clinical benefit/risk analysis the number-needed-to-treat in VTE risk subgroups was calculated as the inverse of the absolute risk reduction, assuming a 50% reduction in the relative risk of VTE with primary prophylaxis. Conversely, we assumed a 25% relative increase in the risk of major and clinically relevant non-major bleeding to calculate the number-needed-to-harm as the inverse of the absolute increase with primary prophylaxis of VTE [25].

## Results

### Patient characteristics

Six-hundred-fifty-seven TGCT patients were identified at the Medical University of Graz (‘Graz Cohort’) and 349 TGCT patients at the University Hospital Zurich for the validation cohort (‘Zurich cohort’). The cohorts were well matched and proportions of the IGCCCG risk groups were consistent with the literature (Table 1) [14,26]. All patients with metastatic disease received cisplatin based chemotherapy. Baseline characteristics of the Graz cohort are listed in Table 2. In the Graz cohort 22 out of 657 (3.3%) and in the Zurich cohort 7 out of 349 (2.0%) patients received primary thromboprophylaxis with low molecular weight heparin for the duration of their chemotherapy. Prophylaxis was prescribed at the individual physician’s discretion. Vitamin K antagonists and new oral anticoagulants were not used. Of those prescribed thromboprophylaxis, 2 out of 22 patients in the Graz cohort and 3 out of 7 patients in the Zurich cohort suffered from VTE. To rule out bias by not excluding these small numbers of patients with thromboprophylaxis a sensitivity analysis was performed. Sensitivity analyses showed that some measures tended to be even stronger after exclusion of patients with primary thromboprophylaxis (Harell’s C coefficient including all patients in the Graz cohort was 0.75, and 0.76 after excluding these patients. The univariable hazard ratio for large retroperitoneal lymphadenopathy in the Graz cohort was 6.8 including all patients, and 8.3 excluding the 22 patients).

**Table 1:** Patient clinical characteristics.

		University of Graz		University of Zurich	
		N = 657		N = 349	
		Number (%missing)	Percentage	Number (%missing)	Percentage
Median Age, years		35.9		34.9	
Histology		(1.4%)		(0.6%)	
	Seminoma	388	59.9	197	56.8
	Nonseminoma	260	40.1	150	43.2
Clinical tumor stage		(2.6%)		(0.0%)	
	Stage IA-B	463	72.3	226	64.8
	Stage IS	9	1.4	9	2.6
	Stage IIA-IIC	98	15.3	50	14.3
	Stage IIIA-C	70	10.9	64	18.3
IGCCCG risk group		(0.0%)		(7.3%)	
	Good	137	76.1	77	67.6
	Intermediate	19	10.6	21	18.4
	Poor	24	13.3	16	14.0
VTE events		34	5.2	18	5.2
Primary Thromboprophylaxis		22	3.3	7	2.0

IGCCCG, International Germ Cell Cancer Collaborative Group; VTE, venous thromboembolism.

**Table 2:** Baseline characteristics of the Graz cohort—Distribution overall and by VTE status.

Variable		Subjects with available data {%missing}	Overall Graz cohort (n = 657)	VTE during follow-up (n = 34)	No VTE during follow-up (n = 623)	P*
**Demographic characteristics**						
Age, years		657 {0.0%}	35.9 [29.2–43.0]	36.5 [27.7–40.1]	35.7 [29.2–43.1]	0.5
BMI, kg/m²		634 {3.5%}	24.7 [22.8–27.2]	23.9 [21.8–26.3]	24.8 [22.9–27.4]	0.11
Family history of TGCT**		463 {29.5%}	17 (3.7%)	0 (0.0%)	17 (3.8%)	0.39
Smoker or Ex-Smoker		555 {15.5%}	281 (50.6%)	11 (50.0%)	270 (50.7%)	0.95
Karnofsky Index <100%		647 {1.5%}	66 (10.2%)	10 (30.3%)	56 (9.1%)	<0.0001
**Clinicopathological variables**						
Non-Seminomatous histology		648 {1.4%}	260 (40.1%)	22 (66.7%)	238 (38.7%)	0.001
Clinical tumor stage		640 {2.6%}				<0.0001
	stage IA-IB		463 (72.3%)	10 (2.2%)	453 (97.8%)	
	stage IS		9 (1.4%)	1 (11.1%)	8 (88.9%)	
	stage IIA–IIC		98 (15.3%)	8 (8.2%)	90 (91.8%)	
	stage IIIA–IIIC		70 (10.9%)	15 (21.4%)	55 (78.6%)	
RPLN(>5cm)		652 {0.8%}	50 (7.7%)	11 (22.0%)	39 (78.0%)	<0.0001
IGCCCG risk stratification		180 {0.0%}				0.004
	Good risk		137 (76.1%)	13 (9.5%)	124 (90.5%)	
	Intermediate risk		19 (10.6%)	7 (36.8%)	12 (63.2%)	
	Poor risk		24 (13.3%)	4 (16.7%)	20 (83.3)	
Chemotherapy cycles		653 {0.6%}				<0.0001
	0 cycles		367 (56.2%)	4 (1.1%)	363 (98.9%)	
	1 cycle		37 (5.7%)	0 (0.0%)	37 (100%)	
	2 cycles		91 (13.9%)	6 (6.6%)	85 (93.4%)	
	3 cycles		105 (16.1%)	10 (9.5%)	95 (90.5%)	
	≥4 cycles		53 (8.1%)	14 (26.4%)	39 (73.6%)	
**Laboratory parameters**						
Hemoglobin, g/dL (13-17.5)		464	15.4 [14.7 1–16.2]	15.6 [14.6–16.1]	15.4 [14.7–16.2]	0.93
WBC, G/L (4.4–11.3)		461	7.7 [6.2–9.5]	8.2 [6.0–9.3]	7.7 [6.2–9.5]	0.93
Platelet count, G/L (140–440)		461	231 [199–273]	226 [191–274]	232 [201–273]	0.59
CRP, mg/L (≤ 5)		427	1.8 [1.0–7.7]	6.7 [2.4–51.0]	1.8 [1.0–6.3]	0.004
Fibrinogen, mg/dL (210 –400)		405	313 [250–425]	410 [324–653]	309 [249–418]	0.003
Tumor markers						
	Preoperative AFP, ng/mL (≤ 15)	581	5.2 [3.0–12.0]	14.0 [3.3–517.7]	5.0 [3.0–10.1]	0.008
	Preoperative betaHCG, mU/mL (≤ 5)	592	5.0 [2.0–11.2]	6.1 [2.0–48.5]	5.0 [2.0–9.4]	0.12
	Preoperative LDH, U/L (120-240)	474	216 [178–295]	343 [237–800]	212 [175–283]	<0.0001
**Khorana Score**		586				0.002
	Score = 1		502 (85.7%)	20 (4.0%)	482 (96.0%)	
	Score = 2		75 (12.8%)	10 (13.3%)	65 (86.7%)	
	Score = 3		9 (1.5%)	0 (0.0%)	9 (100%)	
**Follow-up data**						
	Recurrence of cancer	657 {0.0%}	63 (9.6%)	10 (29.4%)	53 (8.5%)	< 0.0001
	Death	655 {0.3%}	19 (2.9%)	3 (8.8%)	16 (2.6%)	< 0.04
	Median follow up	657 {0.0%}	6.6 [9.7–3.3]			

Continuous data are reported as medians with 25^th^ percentile– 75^th^ percentile in the squared brackets, categorical data are reported as absolute frequencies and percentages in parentheses. Percentages are calculated by referring only to the patients without missing values (i.e. not to the total number of patients if missing values are present). VTE, venous thromboembolism; BMI, Body Mass Index; TGCT, testicular germ cell tumor; RPLN, retroperitoneal lymphadenopathy; IGCCCG, International Germ Cell Cancer Collaborative Group; WBC, white blood count; CRP, C-reactive protein; AFP, alpha Fetoprotein; betaHCG, beta Human Choriogonadotropin; LDH, Lactate dehydrogenase.

*p represents test for difference between VTE and No VTE (χ^2^ tests for binary and categorical variables, ranksum-tests for continuous variables),

**Family history is defined as a history of testicular cancer in a first and/or second degree relative;

### Cumulative risk of VTE in the Graz cohort

Over a median follow-up of 6.6 years (range: 21 days– 14.7 years), 34 VTE events (5.2%) occurred in 657 patients. The cumulative 3-month, 6-month, 12-month, 24-month, and 5-year incidence of VTE accounting for death as a competing risk was 3.7% (95%CI: 2.4–5.3), 4.1% (95%CI: 2.8–5.8), 4.8% (95%CI: 3.3–6.6), 4.9% (95%CI: 3.4–6.8), and 5.3% (95%CI: 3.7–7.2). The most frequent type of VTE event was PE, followed by DVT (Table 3).

**Table 3:** Overall incidence of Venous thromboembolic events (Graz cohort).

Type of thromboembolic event	No. of Patients (N = 657)
All	34 (5.2%)
DVT alone	8 (23.5%)
PE alone	20 (58.8%)
DVT and PE	5 (14.7%)
Visceral TE	1 (2.9%)
**Symptomatic**	22 (64.7%)
**Incidental**	12 (35.3%)
**Fatal**	1 (2.9%)

TE, thromboembolism; DVT, deep venous thrombosis; PE, pulmonary embolism.

### Impact of VTE on mortality in the Graz cohort

The cumulative risk of mortality in the overall study population was 3.6% (95%CI: 2.3–5.6). This risk varied according to clinical stage, with an overall 5-year mortality of 1.0% (0.4–2.7), 3.0% (1.0–8.9), and 16.7% (9.6–28.2) in cS I, cS II and cS III disease, respectively (p<0.0001). In a unidirectional multi-state model, the onset of VTE was associated with a 4-fold increase in the risk of death (transition hazard ratio (THR) = 4.0, 95%CI: 1.2–13.8, p = 0.03). However, this association did not persist after adjusting for tumor stage (adjusted THR for VTE = 1.2, 95%CI: 0.3–4.2, p = 0.82), suggesting that the adverse univariable association between VTE and an unfavourable survival experience is confounded by the strong association between higher tumor stage and higher mortality. In a landmark analysis, patients that experienced VTE within the landmark date 3 months after baseline had a significantly worse 5 year survival than patients who did not develop VTE (92.6% vs 97.7%, Mantel-Byar p = 0.01).

### Predictors of VTE in the Graz cohort

Because VTEs almost exclusively occurred during the first year of follow-up, we restricted the following time-to-event analyses to a 1-year time interval. In univariable competing risk analysis, performance status, higher tumor stage, chemotherapy, non-seminomatous histology, large RPLN, higher IGCCCG risk classification, elevated tumor markers, CRP, fibrinogen and elevated Khorana score (i.e. > the 1 point assigned for testicular cancer) were significantly associated with an increased one-year risk of VTE (Table 4). The two strongest predictors for an increased risk of VTE were stage (χ^2^ on 2 degrees of freedom = 36.5). and chemotherapy (χ^2^ on 1 degree of freedom = 16.7). Therefore, multivariable adjustment for these variables was performed (Table 4). Due to the strong collinearity between stage and chemotherapy leading to model instability upon inclusion of both of these variables, this adjustment was done separately. For subsequent multivariable analyses, we only considered stage because this variable showed a stronger association with (see χ^2^ above) and a slightly higher discriminatory potential (ROC-AUC = 0.77 vs. ROC-AUC = 0.76) towards VTE than chemotherapy, respectively.

**Table 4:** Baseline parameters and one-year risk of VTE in TGCT patients (Graz cohort)—Uni—And multivariable competing risk regression (Fine & Gray proportional hazards model).

	Univariable analysis			Multivariable analysis adjusted for tumor stage			Multivariable analysis adjusted for chemotherapy		
Variable	SHR	95%CI	p	SHR	95%CI	P	SHR	95%CI	p
**Demographic characteristics**									
Age (per 5 year increase)	0.88	0.74–1.05	0.15	0.91	0.77–1.07	0.27	0.97	0.83–1.14	0.73
BMI (per 5kg/m^2^ increase)	0.70	0.40–1.22	0.21	0.81	0.51–1.29	0.37	0.74	0.45–1.24	0.26
Family history	N/A			N/A			N/A		
Smoker or Ex-Smoker	0.97	0.40–2.33	0.95	0.86	0.35–2.12	0.76	0.79	0.33–1.90	0.60
Karnofsky Index <100%	4.65	2.18–9.93	<0.0001	2.24	0.97–5.24	0.06	3.45	1.59–7.51	0.002
**Clinical variables**									
Non-Seminomatous histology	4.23	1.88–9.49	<0.0001	2.29	0.84–6.25	0.11	1.61	0.67–3.88	0.29
Clinical tumor stage									
---stage IA-IB (reference category)									
---stage IS, IIA-IIC	4.47	1.68–11.9	0.003	N/A			1.97	0.73–5.32	0.18
---stage IIIA-IIIC	13.82	5.88–32.50	<0.0001	N/A			4.87	1.97–12.00	0.001
RPLN (>5cm)	7.81	3.71–16.43	<0.0001	2.11	0.86–5.16	0.10	3.29	1.54–7.05	0.002
IGCCCG risk stratification									
---Good risk (reference category)									
---Not good risk (Intermediate and poor risk)	3.19	1.42–7.21	0.005	N/A			2.61	1.16–5.88	0.02
Chemotherapy	19.59	4.71–81.58	<0.0001	9.27	2.07–41.44	0.004	N/A		
**Laboratory variables**									
Preoperative									
---Hemoglobin (per 1g/dL increase)	0.92	0.70–1.21	0.54	1.09	0.87–1.35	0.46	0.95	0.74–1.22	0.68
---WBC (per 1G/L increase)	1.04	0.88–1.23	0.62	1.01	0.87–1.18	0.88	0.99	0.83–1.19	0.91
---Platelet count (per 50G/L increase)	1.06	0.70–1.61	0.78	0.90	0.69–1.17	0.43	0.93	0.63–1.36	0.70
---CRP (per 1log increase)	1.47	1.15–1.88	0.002	1.04	0.79–1.36	0.81	1.26	0.98–1.63	0.07
---Fibrinogen (per 100mg/dL increase)	1.36	1.13–1.64	0.001	1.00	0.81–1.25	0.97	1.18	0.96–1.45	0.11
Tumor markers (preoperative)									
---AFP (per 1log increase)	1.36	1.20–1.54	<0.0001	1.18	1.00–1.38	0.05	1.22	1.07–1.39	0.003
betaHCG (per 1log increase)	1.21	1.07–1.37	0.003	1.05	0.91–1.20	0.52	1.09	0.95–1.26	0.21
LDH (per 1log increase)	3.27	2.11–5.08	<0.0001	1.86	1.06–3.25	0.03	2.37	1.48–3.80	<0.0001
**Khorana Score**									
Score = 1 (reference category)									
Score≥2	3.72	1.70–8.13	0.001	1.91	0.84–4.32	0.12	2.22	1.02–4.85	0.05

VTE, venous thromboembolism; TGCT, testicular germ cell tumor; SHR, subhazard ratio; CI, confidence interval; BMI, Body Mass Index; RPLN, retroperitoneal lymphadenopathy; IGCCCG, International Germ Cell Cancer Collaborative Group; WBC, white blood count; CRP, C-reactive protein; AFP, alpha Fetoprotein; ß-HCG, beta Human Choriogonadotropin; LDH, Lactate dehydrogenase.

In a subgroup analysis of only patients with metastasized cS IS–IIIC disease (n = 177, i. e. a population that corresponds to the development cohort of the Srikanthan model), the most relevant predictors of VTE were clinical stage III disease, a large RPLN and intermediate/poor IGCCCG risk (Table 5). In a subgroup analysis of only stage IIIA-C patients (n = 70), a large RPLN did not emerge as a significant predictor of VTE risk (SHR = 1.84, 95%CI: 0.65–5.23, p = 0.25) suggesting that other contributing factors (greater burden of disease) increase the risk of VTE.

**Table 5:** Subgroup analysis: Baseline parameters and the one-year risk of VTE in TGCT patients with non-metastasized disease and metastasized disease—–Fine & Gray competing risk regression.

	Univariable analysis in cS IA-IB (463 patients)			Univariable analysis in cS IS-IIIC (177 patients)		
Variable	SHR	95%CI	P	SHR	95%CI	P
**Demographic characteristics**						
Age (per 5 year increase)	1.14	0.89–1.44	0.29	0.83	0.68–1.00	0.05
BMI (per 5kg/m^2^ increase)	1.19	0.54–2.63	0.67	0.66	0.37–1.18	0.16
Family history	N/A			N/A		
Smoker or Ex-Smoker	1.02	0.21–5.04	0.98	0.91	0.32–2.58	0.86
Karnofsky Index < 100%	2.23	0.28–17.42	0.45	2.61	1.12–6.09	0.03
**Clinicopathological variables**						
Non-Seminomatous histology	14.92	1.85–120.42	0.01	1.57	0.64–3.86	0.32
Clinical tumor stage						
---stage IA—IB (reference category)	N/A			N/A		
---stage IS, IIA—IIC	N/A			stage IS—IIC (reference category)		
---stage IIIA—IIIC	N/A			3.08	1.31–7.27	0.01
RPLN (>5cm)	N/A			2.71	1.18–6.24	0.02
IGCCCG risk stratification						
---Good risk (reference category)						
---Not good risk (Intermediate and poor risk)	N/A			3.12	1.38–7.04	0.006
Chemotherapy	7.84	1.60–38.50	0.01	4.20x10^32^*	N/A	N/A
Radiotherapy	N/A			6.76x10^-20^*	N/A	N/A
Laboratory parameters						
Preoperative						
---Hemoglobin (per 1g/dL increase)	1.17	0.67–2.06	0.57	1.05	0.76–1.45	0.77
---WBC (per 1G/L increase)	0.96	0.79–1.17	0.69	1.06	0.85–1.32	0.62
---Platelet count (per 50G/L increase)	0.91	0.61–1.38	0.67	0.93	0.63–1.37	0.71
---CRP (per 1log increase)	1.03	0.80–1.33	0.81	1.15	0.84–1.57	0.39
---Fibrinogen (per 100mg/dL increase)	0.92	0.63–1.35	0.67	1.13	0.89–1.44	0.33
Preoperative AFP (per 1log increase)	1.45	1.13–1.86	0.004	1.17	1.00–1.36	0.05
Preoperative betaHCG (per 1log increase)	1.07	0.61–1.89	0.80	1.10	0.96–1.26	0.19
Preoperative LDH (per 1log increase)	2.89	0.99–8.44	0.05	2.03	1.17–3.53	0.01
Khorana Score						
Score = 1 (reference category)						
Score≥2	N/A			2.66	1.08–6.53	0.03

VTE, venous thromboembolism; TGCT, testicular germ cell tumor; BMI, Body Mass Index; RPLN, retroperitoneal lymphadenopathy; IGCCCG, International Germ Cell Cancer Collaborative Group; LDH, Lactate dehydrogenase; WBC, white blood count; CRP, C-reactive protein; AFP, alpha Fetoprotein; ß-HCG, beta Human Choriogonadotropin;

*these HR are extremely high or extremely small, which is indicative of model instability; this is because VTE events were almost exclusively clustered in patients who received chemotherapy, whereas the very few patients that received radiotherapy were stage II seminomas.

In a subgroup analysis of patients with non-metastasized cS IA-B disease, we found that adjuvant chemotherapy was associated with a 4-fold increase in the risk of VTE when compared to those cS IA-B patients without adjuvant chemotherapy (Hazard Ratio = 4.0, 95%CI: 1.1–13.9, p = 0.03) (Table 5).

### A stratification for 12-month risk of TGCT-associated VTE

**Fig 1:**
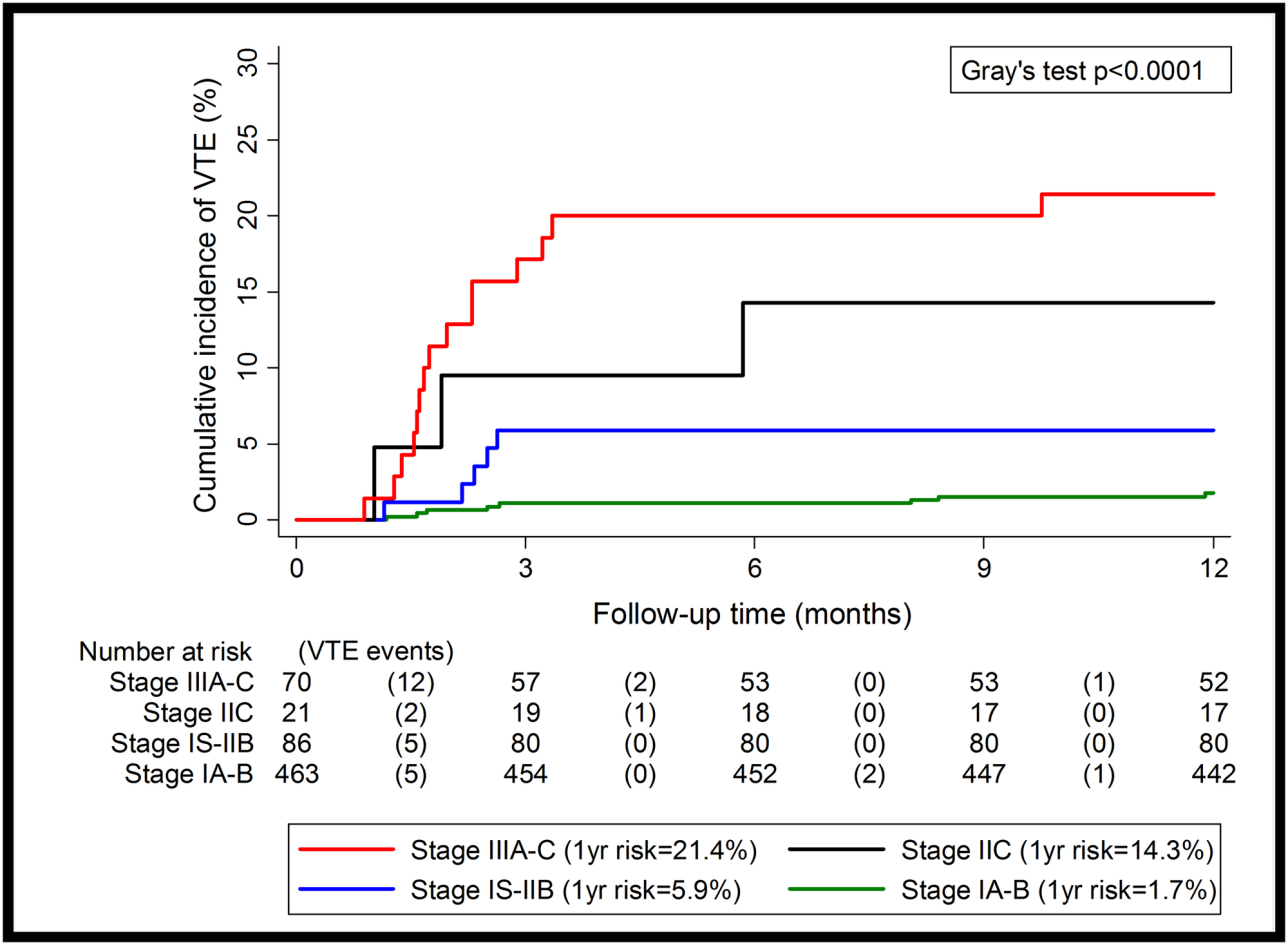
12-month-cumulative VTE incidence in the Graz cohort.

In our model, higher tumor stage and a large RPLN where the main risk factors for VTE in patients with metastasized disease. As the presence of a RPLN with more than 5cm in greatest dimension without coexisting visceral metastases is classified as stage IIC, we constructed an empirical VTE risk stratification rule with the following four categories: cS IA-B (12-month VTE risk: 1.7%), cS IS-IIB (5.9%), cS IIC (14.3%), and cS IIIA-C (21.4%) (Fig 1). In competing risk regression, we observed increasing relative risks of VTE according to this rule (cS IA-B: reference category; cS IS-IIB: SHR = 3.45, 95%CI: 1.13–10.53, p = 0.03; cS IIC: SHR = 8.86, 95%CI: 2.35–33.45, p = 0.001; cS IIIA-C: SHR = 13.82, 95%CI: 5.88–32.51, p<0.0001). The rule discriminated well between patients who did and did not develop VTE during the first 12-months of follow-up (Harell’s C Index = 0.77). In comparative ROC analysis, we observed that a classifier according to these 4 categories had a significantly higher discriminatory performance with respect to VTE than the RPLN-VTE-risk-classifier according to Srikanthan et al. (AUC-ROC: 0.75 vs. 0.63, p for difference = 0.007).

### External validation

**Fig 2:**
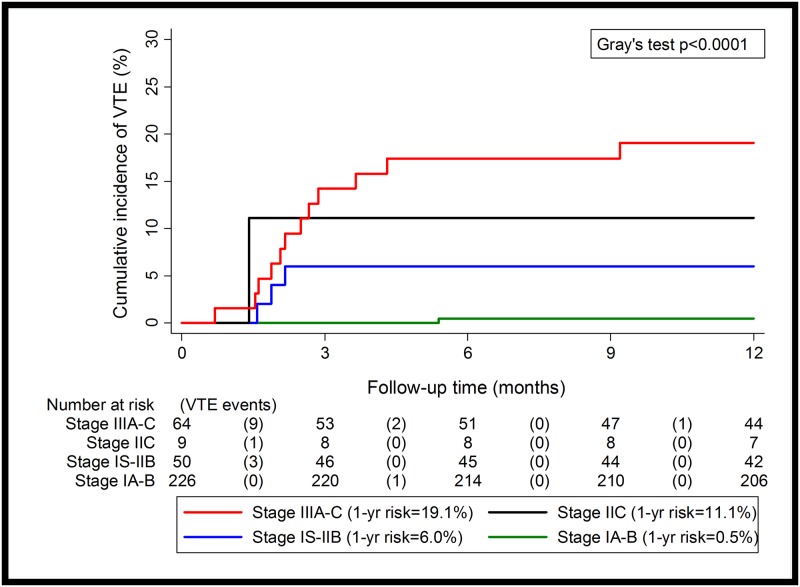
12-month-cumulative VTE incidence in the Zurich cohort.

In the Zurich cohort comprising 349 TGCT patients [Stage IA-B: n = 226 (64.8%), Stage IS-IIB: n = 50 (14.3%), Stage IIC: n = 9 (2.6%), Stage IIIA-IIIC: n = 64 (18.3%)], we observed 18 VTE (5.2%) events. In a competing risk analysis the cumulative one-year incidence of VTE according to our proposed risk stratification categories was highly similar to our development cohort (Fig 2, Table 6). Also, the C-Index for 12-month VTE discrimination was even higher than in the Graz cohort (Harell’s C = 0.84). These findings are consistent with the concept that this score validates in a large external cohort, and represents a valid risk stratification model for VTE in TGCT patients (Table 6).

**Table 6:** Externally validated risk stratification model for VTE in TGCT patients.

Risk category	12 month VTE risk (Graz cohort)	12 month VTE risk (Zurich cohort)
Stage IA—IB	2%	1%
Stage IS—IIB	6%	6%
Stage IIC	14%	11%
Stage IIIA—IIIC	21%	19%

In a sensitivity analysis, we re-fitted the risk stratification rule in the Graz and Zurich cohorts with a prediction time horizon of 3 and 6 months instead of 12-months. The risk stratification rule featured a very high discriminatory potential for these horizons as well (C-Index for 3-month VTE risk in the Graz cohort = 0.79, for 6-month VTE risk in the Graz cohort = 0.81, for 3-month VTE risk in the Zurich cohort = 0.86, and for 6-month VTE risk in the Zurich cohort = 0.84), which these C-Indices being comparable to the one observed with a 12-month horizon.

In a further analysis, we compared our risk rule with the Srikanthan model in the Zurich cohort. In concordance with the Graz cohort, the risk rule also here discriminated better according to future VTE status than the Srikanthan model (ROC-AUC = 0.88 vs. 0.76, p = 0.04).

Moreover, we compared the herein proposed risk rule with the Srikathan model in the Graz cohort, but restricted the population to patients that received chemotherapy for metastastic TGCT (i.e. cS IS-IIIC, n = 177, i.e. a population that is comparable to the population on which the Srikanthan model was developed). Here, the risk stratification had a higher AUC-ROC for VTE (0.65) than the Srikanthan model (0.61), however, this was not statistically significant at the 5% level (p = 0.50). However, in this subgroup of patients the Srikanthan model did not reach significance with respect to discrimination than chance, i. e. the 95% CI of the AUC-ROC includes 0.5 (AUC-ROC = 0.61, 95% CI 0.498–0.757). Our proposed risk stratification rule, showed a significant discriminatory capability as well in the Graz cohort (AUC-ROC = 0.65, 95%CI: 0.56–0.79) as well as in the Zurich cohort (AUC-ROC = 0.68, 95%CI: 0.51–0.78).

### Exploring the clinical risk/benefit-ratio of primary prophylaxis of VTE in TGCT patients

Assuming a 50% relative reduction of the risk of VTE with primary prophylaxis, the numbers-needed-to-treat to prevent one VTE event were 118, 34, 14 and 9 in patients with cS IA-B, cS IS-IIB, cS IIC, cS IIIA-C respectively [25]. Assuming a 25% increase in the relative risk of major and clinically relevant non-major bleeding with primary prophylaxis of VTE, the numbers-needed-to-harm was 125 [25].

## Discussion

In our study population of 657 TGCT patients we have observed an unevenly distributed VTE risk. In stage I TGCT patients the risk of VTE is low, particularly in patients who do not undergo adjuvant chemotherapy. In contrast, TGCT subgroups with a large RPLN and/or distant metastases have a very high risk of VTE. This raises the question whether these patients might benefit from primary prophylaxis.

In unselected patients, the incidence of VTE was 5.2% in the Graz and in the Zurich cohort. In patients with metastatic disease 24 (13.6%) out of 177 patients in the Graz cohort developed a VTE. This again correlates with the VTE rate in the validation cohort of 13.0%.

We confirmed that a large RPLN emerged as a strong risk factor validating the results by Srikanthan et al.[13]. However, in our study population the VTE risk in patients with distant metastases was even higher than in patients with a large RPLN. This observation was also validated in the Zurich cohort and may reflect the increased risk of VTE in a more advanced stage of malignancy with presence of greater burden of disease.

In univariable analysis, we could also confirm that the Khorana score identifies patients with elevated risk for VTE. However, only 9 patients out of our total 657 fell into the high risk category with a Khorana Score ≥ 3, but none of them developed a VTE event, suggesting that the Khorana score may be less useful. Therefore performance of the model was compared with the TGCT-specific model of Srikathan et al. and not the Khorana score. Univariable modeling results indicated an adverse impact of RPLN on VTE risk. This finding is highly consistent with the results of Srikanthan et al. We further showed that our proposed risk stratification rule provides a higher discriminatory accuracy in the risk assessment for VTE than the Srikanthan model in the whole population [13]. In patients with metastatic disease, our proposed rule was numerically but not statistically significantly better with respect to discrimination than the Srikanthan model. However, in this subgroup of patients the Srikanthan model did not reach significance with respect to discrimination than chance, given the 95% CI of the AUC-ROC includes 0.5. This is an interesting finding because the Srikanthan model had also not reached statistical significance in their validation cohort (AUC-ROC = 0.61, 95%CI: 0.43–0.80)[13], whereas our model validates in its external validation cohort from Zurich.

In patients with metastasized cS IS-IIIC disease, undergoing curative chemotherapy, higher tumor stage and a large RPLN were the main risk factors for VTE. Therefore these two predictors built the basis for our risk stratification. We also found some highly significant univariable associations between the properative tumor markers LDH, betaHCG, AFP and VTE risk. However, except for LDH, these differences became much weaker / lost their statistical significance after adjusting for tumor stage. This means that elevations of the tumor markers likely reflect higher tumor stages, which are associated with a high VTE risk.

No thromboembolic events occurred between orchiectomy and the start of chemotherapy. In our observation, the risk of VTE increased immediately after the initiation of chemotherapy which can be explained by cisplatin-induced vascular toxicity [7,27]. Only a negligible amount of events occurred after treatment had been completed. Furthermore, the risk of VTE increased with the number of cycles of chemotherapy, suggesting a dose-dependent risk.

The distribution of TGCT patients according to tumor stage and the incidence of VTE were highly similar in the Graz cohort and the validation cohort from Zurich. Most of TGCT patients present with cS I disease. According to our analysis their VTE risk was low and primary prophylaxis of VTE will have an unfavorable risk-benefit ratio. Conversely, patients with cS IIC and cS III disease carry a very high risk of VTE according to our risk classification. Therefore the numbers-needed-to-treat to prevent one VTE event are relatively low (cS IIC: 14 treated patients, C III: 9 treated patients) based upon relative risk reductions of 50% as shown in prior studies [25]. There is evidence showing that primary prophylaxis works in all (Khorana) risk groups. However, selecting high risk groups may select a population whose risk-benefit ratio with primary prophylaxis will be more favourable. Two reported randomized phase III trials focused on primary prophylaxis of VTE among unselected cancer outpatients treated with chemotherapy and demonstrated a significant reduction in the rate of VTE with primary prophylaxis [25,28]. In the SAVE-ONCO study semuloparin, an ultra-low-molecular-weight heparin, showed a 64% relative risk reduction when compared with placebo [25]. The approval of Semuloparin for prophylaxis of VTE in ambulatory cancer patients was rejected in the United States because of the low event rate of VTE leading to a high number-needed-to-treat and unfavorable risk-benefit profile in an unselected cancer population. Current guidelines thus do not recommend routine prophylaxis of VTE in the outpatient setting except for patients with multiple myeloma receiving thalidomide-lenalidomide-based treatments [29,30]. However, both trials did not include patients with TGCT who have a higher risk. In the Graz cohort the cumulative incidence of VTE was 21% in cS III and 14% in cS IIC patients. In the Zurich cohort these figures were similar with corresponding risks of 19% and 11%. This compares with an incidence of VTE between 17% and 26% in myeloma patients treated with thalidomide or lenalidomide without thromboprophylaxis [31]. In summary, this allows us to speculate that TGCT patients are a cancer population which may have a benefit from primary thromboprophylaxis.

Strengths of this manuscript include a large sample size and an external validation in a large cohort from a different country which yielded highly similar results to the development cohort. Another important strength of our study and the proposed risk model is that this data are applicable to the full spectrum of TGCT patients (and not only to the metastatic setting as investigated by Srikanthan et al.). Further, we could show that the proposed risk stratification rule provides a better discrimination than the Srikanthan model. Moreover, we found that not only RPLN as demonstrated by Srikanthan is a strong risk factor for VTE, but that in addition, stage III disease adds further prognostic information beyond this variable. The Srikanthan model, which was designed specifically for the TGCT setting, already showed better discrimination than the Khorana score. Synoptically, this is consistent with the assumption that to date, our risk stratification rule provides a superior tool for stratifying TGCT patients according to their VTE risk.

The major limitation of the present analysis is its retrospective data collection. Some VTE events may have been missed due to incomplete documentation. This also applies to ascertainment of the primary thromboprophylaxis status. Another limitation is that some VTE events may have occurred outside of our hospital network, which may have led to an underestimation of VTE risk. However, given the observation that most VTEs occur during antineoplastic therapy (where patients are routinely seen at our department) and records of nearly all hospitals in our referral area were accessible by the retrospective data collection team (joint public hospital trust with common IT system and electronic healthcare database), we consider the probability of a VTE rate underestimation to be minimal.

## Conclusions

We demonstrated that TGCT subgroups with large retroperitoneal disease and/or distant metastases carry a very high risk for VTE. These subgroups can be identified with an externally-validated risk assessment model based on the clinical tumor stage, and may benefit from thromboprophylaxis and other management strategies. The efficacy and safety of this approach, as well as optimal thresholds to justify primary prophylaxis of VTE, warrant prospective investigation. In the absence of prospective data, this study supports the concept that that primary prophylaxis of VTE may be worthwhile in patients with cS IIC and cS III TGCT for the duration of their chemotherapy.
